# The COVID‐19 epidemic in Madagascar: clinical description and laboratory results of the first wave, march‐september 2020

**DOI:** 10.1111/irv.12845

**Published:** 2021-02-15

**Authors:** Rindra Vatosoa Randremanana, Soa‐Fy Andriamandimby, Jean Marius Rakotondramanga, Norosoa Harline Razanajatovo, Reziky Tiandraza Mangahasimbola, Tsiry Hasina Randriambolamanantsoa, Hafaliana Christian Ranaivoson, Harinirina Aina Rabemananjara, Iony Razanajatovo, Richter Razafindratsimandresy, Joelinotahiana Hasina Rabarison, Cara E. Brook, Fanjasoa Rakotomanana, Roger Mario Rabetombosoa, Helisoa Razafimanjato, Vida Ahyong, Vololoniaina Raharinosy, Vaomalala Raharimanga, Sandratana Jonhson Raharinantoanina, Mirella Malala Randrianarisoa, Barivola Bernardson, Laurence Randrianasolo, Léa Bricette Nirina Randriamampionona, Cristina M. Tato, Joseph L. DeRisi, Philippe Dussart, Manuela Christophère Vololoniaina, Fidiniaina Mamy Randriatsarafara, Zely Arivelo Randriamanantany, Jean‐Michel Heraud

**Affiliations:** ^1^ Epidemiology and Clinical Research Unit Institut Pasteur de Madagascar Antananarivo Madagascar; ^2^ Virology Unit Institut Pasteur de Madagascar Antananarivo Madagascar; ^3^ University of California Berkeley Berkeley CA USA; ^4^ Chan Zuckerberg Biohub San Francisco CA USA; ^5^ Ministry of Public Health Government of the Republic of Madagascar Antananarivo Madagascar; ^6^ Present address: Virology Department Institut Pasteur de Dakar Dakar Senegal

**Keywords:** COVID‐19, epidemiology, madagascar, pandemic, SARS‐CoV‐2, Surveillance

## Abstract

**Background:**

Following the first detection of SARS‐CoV‐2 in passengers arriving from Europe on 19 March 2020, Madagascar took several mitigation measures to limit the spread of the virus in the country.

**Methods:**

Nasopharyngeal and/or oropharyngeal swabs were collected from travellers to Madagascar, suspected SARS‐CoV‐2 cases and contact of confirmed cases. Swabs were tested at the national reference laboratory using real‐time RT‐PCR. Data collected from patients were entered in an electronic database for subsequent statistical analysis. All distribution of laboratory‐confirmed cases were mapped, and six genomes of viruses were fully sequenced.

**Results:**

Overall, 26,415 individuals were tested for SARS‐CoV‐2 between 18 March and 18 September 2020, of whom 21.0% (5,553/26,145) returned positive. Among laboratory‐confirmed SARS‐CoV‐2–positive patients, the median age was 39 years (IQR: 28‐52), and 56.6% (3,311/5,553) were asymptomatic at the time of sampling. The probability of testing positive increased with age with the highest adjusted odds ratio of 2.2 [95% CI: 1.9‐2.5] for individuals aged 49 years and more. Viral strains sequenced belong to clades 19A, 20A and 20B indicative of several independent introduction of viruses.

**Conclusions:**

Our study describes the first wave of the COVID‐19 in Madagascar. Despite early strategies in place Madagascar could not avoid the introduction and spread of the virus. More studies are needed to estimate the true burden of disease and make public health recommendations for a better preparation to another wave.

## 1 INTRODUCTION

In December 2019, a new coronavirus later named SARS‐CoV‐2 emerged in the city of Wuhan (province of Hubei), China, causing deadly pneumonia.^1^, ^2^ Since then, this virus has spread worldwide and the World Health Organizations (WHO) declared coronavirus disease 2019 (COVID‐19), the disease resulting from SARS‐CoV‐2 infection, a global pandemic on 11 March 2020.^3^ Despite many efforts from countries to contain the spread at the national level, the epidemic is still ongoing in many countries, including those in Africa, although the African epidemic has been somewhat blunted in comparison with European countries and other territories.^4^, ^5^ At the time of submission (31 December 2020), COVID‐19 has resulted in more than 83 million cases and 1,8 million deaths worldwide.^5^ In Africa, the number of cases (2,76) and deaths (65 468) represents a small fraction of the global data. With the exception of anosmia and ageusia in some patients, COVID‐19 is non‐specific and similar to many other respiratory viruses.^6^, ^7^ Therefore, laboratory confirmation is required to positively identify a case.

Madagascar is a large island located in the South‐West of the Indian Ocean with an estimated population of about 27 million, most of whom (65%) inhabit rural areas.^8^ International connection through air‐traffic remains limited with fewer than 50 international flights per week and around 500 000 passengers annually.^8^ In order to mitigate the introduction of SARS‐CoV‐2 to Madagascar from patients arriving from affected countries, the Institut Pasteur de Madagascar established a real‐time RT‐PCR detection platform in country as early as 29 January 2020, thanks to technical support from the Hong Kong University—Pasteur Research Pole.^9^

Following an increasing number of cases in Europe and Asia, one of the regions with high volume of travellers, the Malagasy Government screened all incoming international travellers from 12 to 20 March 2020 and eventually decided to close the country to all air‐traffic on 20 March 2020. After the detection of the first SARS‐CoV‐2 case in Madagascar from an incoming traveller on 19 March 2020, other non‐pharmaceutical interventions were adopted, including curfew, stay‐at‐home order, closure of non‐essential businesses and social distancing in order to prevent or limit the spread of the virus in the country.

The objective of the current study was to describe the epidemiology of the first epidemic wave of SARS‐CoV‐2 affecting Madagascar from 18 March to 18 September 2020, and in particular the proportion of asymptomatic positive cases since the national strategy was to test both travellers and contacts regardless of the presence of symptoms ate the time of sampling.

## 2 MATERIALS AND METHODS

### 2.1 Study subject and specimen collection

Specimen were collected from different types of individuals:

‐ Passengers. Following the strategy from the Ministry of Public Health (MPH), all passengers arriving from Europe and China, from 12 to 20 March (2020) were screened for SARS‐CoV‐2 regardless of symptoms at the time of sampling.
‐ Contacts of positive cases regardless of symptoms at the time of sampling. Contacts were defined as anyone who had direct contact or was within 1 metre of a SARS‐CoV‐2–infected person for at least 15 minutes even if that person had no symptoms (household members, other family contacts, visitors, neighbours, colleagues, teachers, co‐workers) according to the MPH case definition based on WHO guidelines.^10^
‐ Suspected SARS‐CoV‐2 cases. After community transmission was demonstrated in one region or locality in Madagascar, all patients visiting hospitals and clinics with symptoms related to COVID‐19 infection were sampled. Additionally, our existing Influenza Surveillance System (ISS) was extended to include monitoring of COVID‐19 based on recommendations from the WHO ^11^, ^12^, ^13^: patients visiting clinics or hospitals within the ISS network were sampled if presenting with influenza‐like Illness (ILI) or severe acute respiratory infection (SARI) as per the revised WHO case definitions.^14^ Patients that presented with solely anosmia and/or ageusia were also considered as COVID‐19 suspected cases. From each suspected case, demographic and clinical information was collected.
‐ We also received a high number of specimens from public clinics that were opened during the epidemic offering free sampling and screening test. Convenient specimens were also received from public and private institutions.

### 2.2 Viral detection

Nasopharyngeal and/or oropharyngeal swabs were taken and were placed into viral transport media and transported at 4°C to the Virology Unit (National Influenza Centre) at the Institut Pasteur de Madagascar (IPM). Specimens were stored at 4°C before nucleic acid extraction and real‐time RT‐PCR processing. Due to the scarcity of reagents available, specimens were tested using different methods upon availability of reagents. Overall, five real‐time RT‐PCR protocols recommended by WHO were used for the detection of the novel coronavirus 2019 ^15^, ^16^: Charité—Universitätsmedizin Berlin,^17^ Hong Kong University,^9^ Da An gene (Da An Gene Co., Ltd. Sun Yat‐sen University, Guangzhou, China), LightMix® SarbecoV E‐gene plus EAV control (TIB Biolmol, Berlin, Germany), and TaqPath™ COVID‐19 Combo kit (Life Technologies Ltd, Paisley, UK). For clinicians in need of rapid results for patients in the emergency care unit/intensive care unit, specimens were tested using Xpert Xpress SARS‐CoV‐2 cartridges (Cepheid, Sunnyvale, CA, USA), a rapid PCR‐based assay. All tests were performed in accordance with the protocols available provided by the WHO ^15^ and manufacturer's instructions for use.

### 2.3 Full Genome sequencing and genomic analysis

Methods for generating full genome sequences from SRAS‐CoV‐2 strains circulating in Madagascar and subsequent genomic analysis are detailed in Supplementary file.

### 2.4 Data management and analyses

The data included in the record form accompanying the biological samples were collected and managed using REDCap electronic data capture tools hosted at IPM.^18^, ^19^ REDCap (Research Electronic Data Capture) is a secure, web‐based software platform designed to support data capture for research studies, providing 1) an intuitive interface for validated data capture; 2) audit trails for tracking data manipulation and export procedures; 3) automated export procedures for seamless data downloads to common statistical packages; and 4) procedures for data integration and interoperability with external sources. In our analyses, all continuous variables are expressed as median with interquartile range (IQR); categorical variables are presented as percentage, subject to a chi‐squared test. All statistical analysis was performed in R ^20^ and at individual level, and *P‐value* < .05 was considered statistically significant. We carried out a mapping of the geographical distribution of confirmed cases according to the health district where the sample collection originated from.

### 2.5 Patient Consent Statement

All data used by this study were from state‐wide surveillance of a notifiable disease and were de‐identified.

## 3 RESULTS

### 3.1 Characteristics of patients and specimens

From 25 January to 15 March 2020, 96 suspected cases were sampled and all tested negative. The vast majority of these suspected cases tested had a travel history in China and particularly originated from the Hubei Province (personal communication). On 16 March 2020, following the increasing number of cases that occurred in Europe and specifically in Italy, Spain and France, the Government took the decision to test all passengers that have arrived in Madagascar since 12 March 2020, from an affected area. The first imported SARS‐CoV‐2 case in Madagascar was then laboratory‐confirmed on 19 March 2020. Thereafter, several imported cases from passengers were detected. The first laboratory‐confirmed cases without a travel history, therefore considered to be community transmission, were detected on 25 March 2020. Although some cases are still being detected in December 2020, our study focuses on the first six months, or the “first wave,” of the pandemic in Madagascar (ie from 18 March to 18 September 2020).

Overall, we received specimens from 26,468 individuals of which 26,415 (99.8%) were tested for SARS‐CoV‐2 (remaining specimens were rejected for non‐conformity). Among individuals tested, 21.0% (5,553/26,415) were positive (Table 1). The median age of patients from whom specimens were collected was 37 years (IQR: 26‐49 years) and 52.9% were male (13,817/26,138) when excluding missing data on sex. The age distribution of patients from whom specimens were collected was different than the age distribution of the overall Malagasy population, with more individuals over 20 years sampled. (22,397/25,928) (Table 1). Most of the individuals sampled (76.0%; 19,718/25,928) and those who tested positive (77.3%; 4,257/5,507) were aged from 20 to 59 years old and positivity rate increased with age (Figure S1). Among SARS‐CoV‐2–confirmed cases, the sex ratio (M/F) was 1.05 (2,826/2,686) (Table 1). The median age of positive patients was 39 years (IQR: 28‐52 years) and ranged from 1 week to 93 years. When looking at passenger, suspected cases and contact of confirmed cases, we found that positivity rates was 11.2% (98/878), 23.5% (3,819/16,219) and 17.6% (1,636/9,318), respectively (Table S1).

**TABLE 1:** Laboratory results of all individual tested at IPM for SARS‐CoV‐2 by gender, age group and occurrence of symptoms

Total	Positive		Negative		Total		*P‐value* *
	5 553	%	20 862	%	26 415	%	
Sex							*.008*
Male	2 826	50.9	10 991	52.7	13 817	52.3	
Female	2 686	48.4	9 635	46.2	12 321	46.6	
*Missing*	*41*	*0.7*	*236*	*1.1*	*277*	*1.0*	
Age (Years)							<.001
0‐4	78	1.4	728	3.5	806	3.1	
5‐14	191	3.4	1 104	5.3	1 295	4.9	
15‐19	268	4.8	1 162	5.6	1 430	5.4	
20‐29	1 116	20.1	4 270	20.5	5 386	20.4	
30‐39	1 131	20.4	4 503	21.6	5 634	21.3	
40‐49	1 142	20.6	3 782	18.1	4 924	18.6	
50‐59	868	15.6	2 906	13.9	3 774	14.3	
>59	713	12.8	1 966	9.4	2 679	10.1	
*Missing*	*46*	*0.8*	*441*	*2.1*	*487*	*1.8*	
Symptomatic							<.001
Yes	2 242	40.4	4 309	20.7	6 551	24.8	
No	3 311	56.6	16 553	79.3	19 864	75.2	

* Pearson's chi‐squared tests were performed (*P*‐values < .05 were considered significant)

### 3.2 Clinical symptoms of patients

We found that 75.2% (19,864/26,415) of patients tested declared no symptom at the time of sampling. The proportion of asymptomatic individuals was 56.6% (3,311/5,553) among laboratory‐confirmed cases (Table 1). The most common symptoms of illness onset among confirmed cases were cough (27.2%), fever (18.7%), weakness (14.7%), runny nose (13.3%) and headache (13.1%) (Table S2; Table 2). In multiple logistic regression, age and the five most common symptoms observed in confirmed cases were associated with SARS‐CoV‐2 positivity. The probability of having a positive RT‐PCR increased with age (Figure S1). Compared to individuals less than 16 years, individuals aged 16 and above had higher probability to have a positive RT‐PCR. The adjusted odds ratios (aOR) were 1.8 [95% CI: 1.6‐2.1]) for individuals aged 16 to 49 years and 2.2 [95% CI: 1.9‐2.5]) for individuals aged 50 years and more. We estimated that, compared to individuals without fever, individuals with fever were two times more likely to have a positive RT‐PCR (aOR_=_1.9 [95% CI: 1.7‐2.1]), while those with cough and weakness were, respectively, 1.8 and 1.4 times more likely to test positive (aOR cough = 1.8 [95% CI: 1.7‐2.0]; aOR weakness = 1.4 [95% CI: 1.3‐1.6]). Those with runny nose and headache had respective aORs of 1.3 [95% CI: 1.1‐1.5] and 1.2 [95% CI: 1.1‐1.3].

**TABLE 2:** Association of RT‐PCR results with age, sex and clinical symptoms. Only data from individuals with no missing information (ie age sex and symptoms) were included

Covariates	RT‐PCR results		OR (95% CI)	aOR (95% CI)	*P*‐value*
	POS (5 472)	NEG (20 268)			
Age (%)					
<16yrs	312 (5.7)	2,006 (9.9)	1	1	
16‐49yrs	3 586 (65.5)	13 422 (66.2)	1.7 (1.5‐1.9)	1.8 (1.6‐2.1)	< 0.001
>49yrs	1 574 (28.8)	4 840 (23.9)	2.1 (1.8‐2.4)	2.2 (1.9‐2.5)	< .001
Sex (%)					
Female	2 672 (48.8)	9 468 (46.7)	1	1	
Male	2 800 (51.2)	10 800 (53.3)	0.9 (0.9‐1.0)	0.9 (0.9‐1.0)	.05
Cough (%)					
No	3 984 (72.8)	17 924 (88.4)	1	1	
Yes	1 488 (27.2)	2 344 (11.6)	2.9(2.7‐3.1)	1.8 (1.7‐2.0)	< .001
Fever (%)					
No	4 447 (81.3)	18 932 (93.4)	1	1	
Yes	1 025 (18.7)	1 336 (6.6)	3.3 (3.0‐3.6)	1.9 (1.7‐2.1)	< .001
Weakness					
No	4 668 (85.3)	19 131 (94.4)	1	1	
Yes	804 (14.7)	1 137 (5.6)	2.9 (2.6‐3.2)	1.4 (1.3‐1.6)	< .001
Runny nose					
No	4 743 (86.7)	18 908 (93.3)	1	1	
Yes	729 (13.3)	1 360 (6.7)	2.1 (1.9‐2.3)	1.2 (1.1‐1.3)	.003
Headache					
No	4 755 (86.9)	19 180 (94.6)	1	1	
Yes	717 (13.1)	1 088 (5.4)	2.7 (2.4‐2.9)	1.3 (1.1‐1.5)	< .001

Abbreviation: OR, Crude Odd ratio; aOR, Adjusted Odd ratio.

* Pearson's chi‐squared tests were performed (*P*‐values < .05 were considered significant).

### 3.3 Circulation of SARS‐CoV‐2 in Madagascar

**FIGURE 1:**
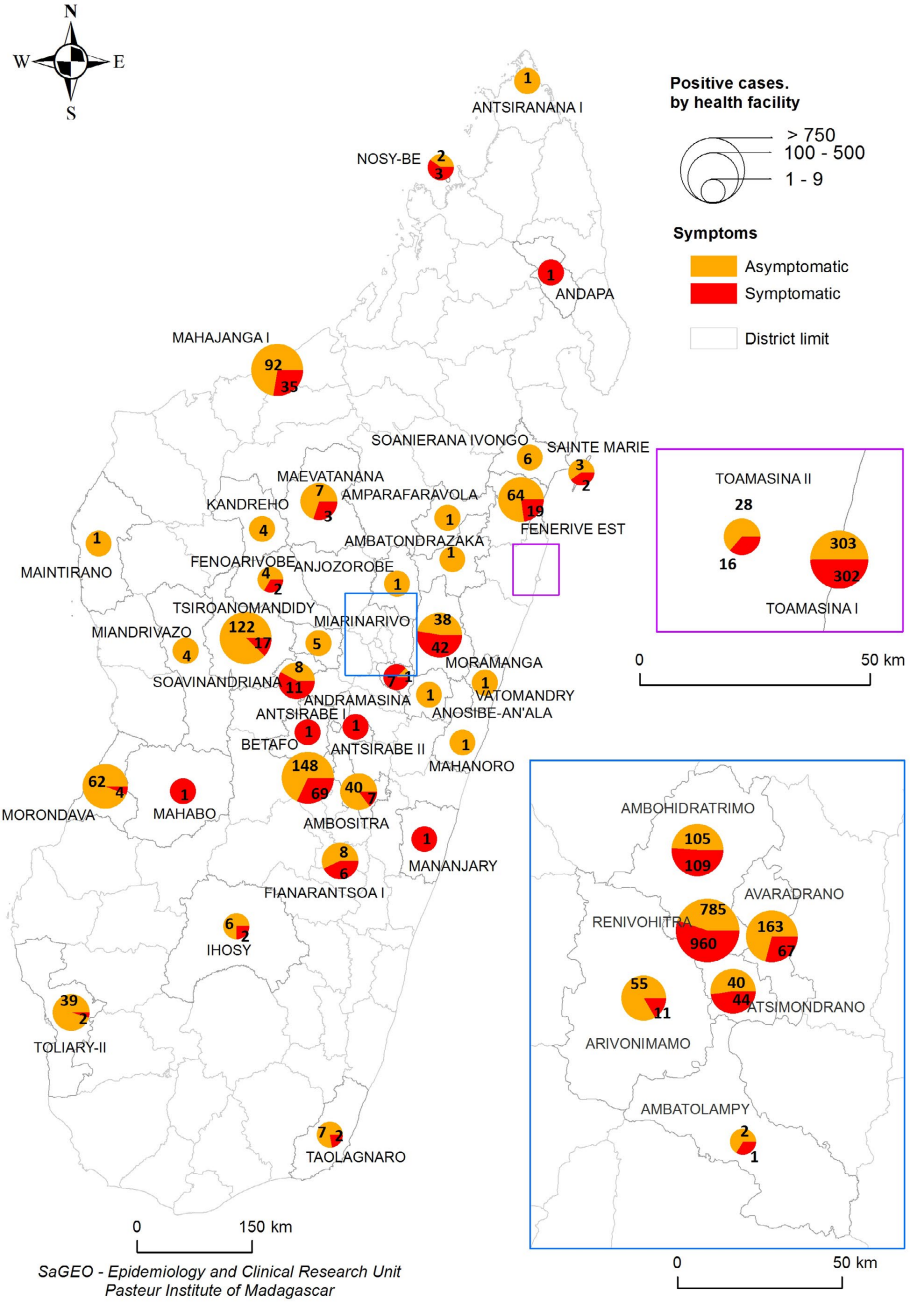
Distribution of positive cases in Madagascar from 18 March to 18 September 2020. Pies shows the numbers of symptomatic (red) and asymptomatic (orange) SARS‐CoV‐2 laboratory‐confirmed cases. Pie size is proportional to the total number of cases per region

**FIGURE 2:**
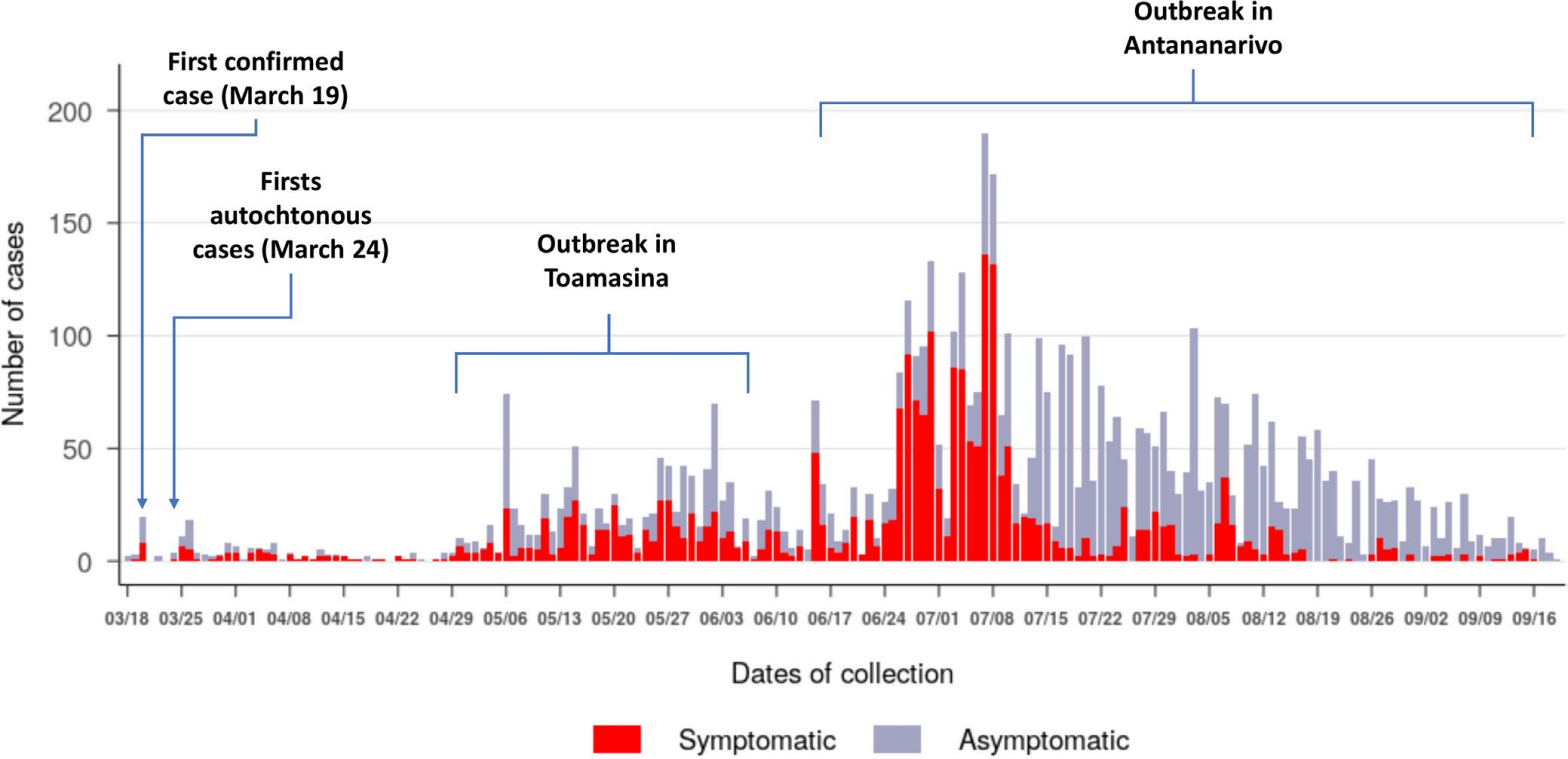
Weekly SARS‐CoV‐2 laboratory‐confirmed cases in Madagascar from 18 March to 18 September 2020. SARS‐CoV‐2–positive cases are represented according symptoms presented at the time of collection (n = 5 553)

**FIGURE 3:**
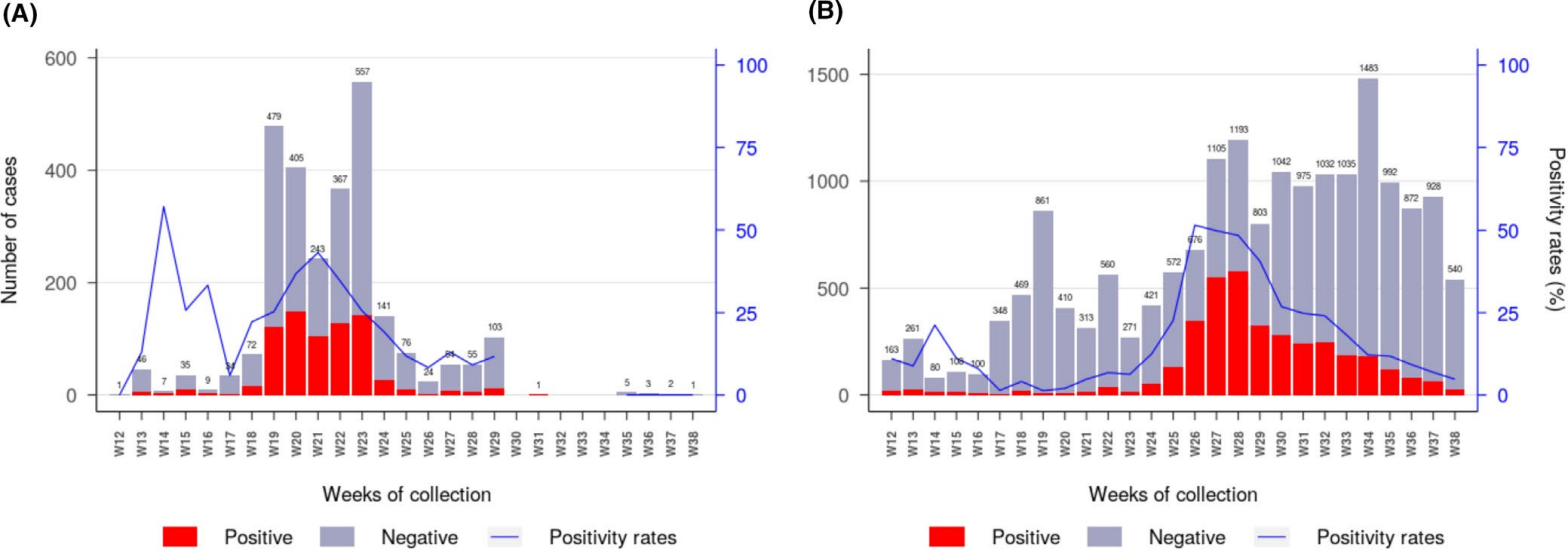
Weekly laboratory results and positivity rate for SARS‐CoV‐2 in Toamasina (A) and Antananarivo (B) regions from week 12 to week 38. For the Toamasina Region, specimens (n = 2 720) originated from two health districts (Toamasina I and Toamasina II). For Antananarivo region, specimens (n = 17 613) originated from five health districts (Andramasina, Ambohidratrimo, Antananarivo‐Avaradrano, Antananarivo‐Atsimondrano, and Antananarivo‐Renivohitra districts). (remark: week 12 started on 16th of March and week 38 ended on 20th of September 2020)

During the first wave of the epidemic, the virus spread in almost all regions of Madagascar (Figure 1). At the national level, active circulation of the virus in the community was observed in a first surge from May to June followed by a second but more intense surge from the end of June to the end of July (Figure 2). These two consecutive peaks were driven by community outbreaks occurring in two highly populated regions of the country (Toamasina and Antananarivo) (Figure 3). The first city affected was Toamasina, located on the East coast, the second most populated city of Madagascar and the main seaport of the country. In this city, sporadic cases were detected from 16 March to 26 April 2020 from individuals with (i) a history of travel in countries with SARS‐CoV‐2 community transmission or (ii) contacts with travellers that tested positive. During week 18 (27 April to 03 May 2020), several clusters of cases were detected, many among the employees of a large mining company. From these clusters, the virus quickly spreads into the community, causing an ensuing outbreak, which lasted for 8 weeks (from 27 April to 21 June 2020) (Figure 3A). During that period, the peak of cases was observed during week 20 (mid‐May). The positivity rate reached 43.2% on week 21 (18‐24 May 2020) and decreased thereafter. The second city affected was Antananarivo, the capital city of Madagascar with around 2.6 million inhabitants. The epidemic started in Antananarivo during week 24 (8‐14 June 2020) (Figure 3B). The epidemic peaked on week 28 (6‐12 July 2020) with the positivity rate of about 50%. Although cases were still detected at the end of our study, the positivity rate was below 10% by end of August and the number of daily cases was below 10 by week 38 (14‐20 September 2020).

### 3.4 Monitoring of COVID‐19 through the Influenza Surveillance System

**FIGURE 4:**
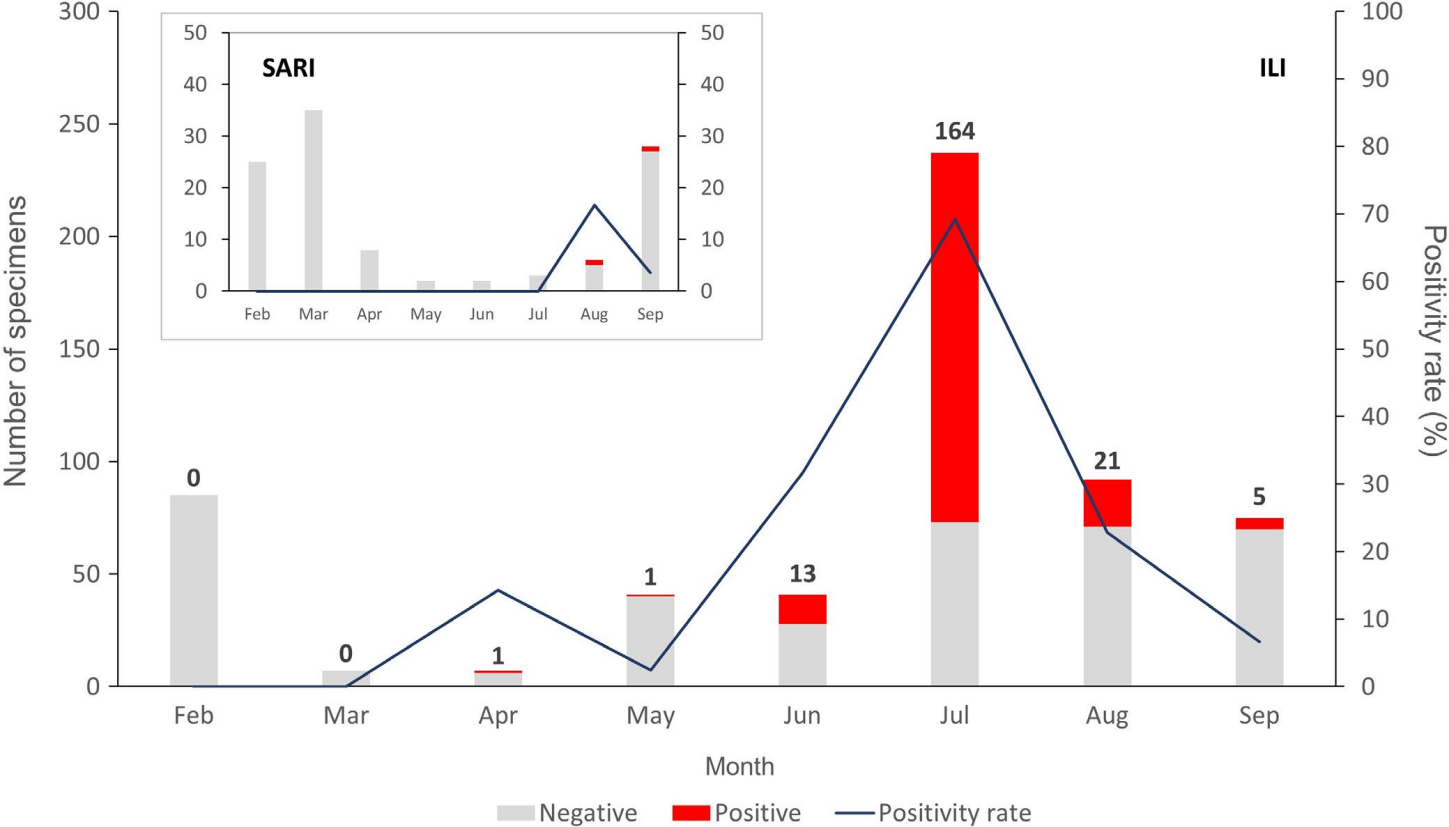
Monthly laboratory results from the Severe Acute Surveillance Infection (SARI) and Influenza‐Like Illness (ILI) surveillance in Madagascar from February to September 2020. Each bar represents the total number of negative cases (grey) and SARS‐CoV‐2–positive cases (red). Numbers above bars indicate the number of positives. The dark blue line represents the positivity rate

A proportion of the overall specimens received during the COVID‐19 epidemic were acquired through the extension of the ISS to include SARS‐CoV‐2. Although our SARI surveillance system only detected a few COVID‐19 cases (with very few samples received from May to July and only two SARI‐derived SARS‐CoV‐2–confirmed cases in August and September), the ILI system sourced a substantial number of COVID‐19–positive samples (Figure 4). Overall, among ILI suspected cases, 35.0% (205/584) of them were found positive for SARS‐CoV‐2. The peak positivity rate reached 69.2% (164/237) in July and decreased thereafter.

### 3.5 Genetic characteristics of the newly introduced SARS‐CoV‐2 virus in Madagascar

The entire genomes of the 10th and 19th cases of SARS‐CoV‐2 detected in Madagascar (from 20 and 22 March 2020) were obtained on an Illumina platform (iSeq100) and deposited in the GISAID EpiCoV™ database (*EPI_ISL_508862|2020‐03‐20, EPI_ISL_508863|2020‐03‐22*). The patients from which both genomes were obtained arrived from France (Paris) on 18 and 19 March 2020. The analysis of the complete genome of both samples revealed a sequence homology of 99.92% when compared to the reference virus originated from Wuhan (hCov‐19/Wuhan/WIV04/2019). These two viruses belong to the clade 20A, lineage B.1 (Figure S2), which was prevalent in Europe at the time of introduction.^21^ Several amino acid substitutions were observed at the following sites: the viral Spike glycoprotein (D614G), accompanied (as is customary to this clade) by a C‐to‐T mutation in the 5′ untranslated region at position 241, a synonymous C‐to‐T mutation at position 3037, a non‐synonymous C‐to‐T mutation at position 14 408 in the RNA‐dependent RNA polymerase gene (ORF1b‐Nsp12:P314L) and a non‐synonymous G‐to‐T mutation at position 25 563 (Orf3a: Q57H) (Table S3). In addition to these common mutations, both early sequences also exhibited C‐to‐T mutations at positions 2416 and 5884.

Two additional samples collected on 26 March 2020 from the beginning of the outbreak in Toamasina were sequenced (EPI_ISL_677635/2020‐03‐26, EPI_ISL_677636/2020‐03‐26) from two mining workers that were also housemate, and who had travelled to Madagascar from the Philippines. These sequences demonstrated a sequence homology of 99.98% when compared to the reference virus (hCov‐19/Wuhan/WIV04/2019); they cluster in a rare Asian subclade within Nextstrain clade 19A (Figure S2), which has been previously described circulating in India with links to Indonesia.^22^ The Toamasina sequences share four mutations with this previously characterized Indian subclade: a non‐synonymous C‐to‐A mutation at position 6312 (Orf1a‐Nsp3: T2016K), the common G‐to‐T mutation at position 11 803 (Orf1a‐Nsp12: L3606F), a non‐synonymous C‐to‐T mutation at position 13 730 (Orf1b‐RdRp: A88V), a C‐to‐T spike protein mutation at position 23 929 and a non‐synonymous C‐to‐T mutation at position 28 311 (N: P13L and Orf9b: P10S) (Table S3). In addition, they also show a C‐to‐T mutation at position 19 524 and a non‐synonymous G‐to‐A mutation at position 1268 (Orf1a‐Nsp2: D335N).

Finally, we have recently begun sequencing samples from later in the Madagascar epidemic, including one sample collected from Toamasina in May (GISAID EPI_ISL_625456/2020‐05‐04) and another from Antananarivo in September (EPI_ISL_677634/2020‐09‐16). Both of these sequences belong to Nextstrain clade 20B, lineage B.1.1 (Figure S2), which is distinguished from the four common mutations that define clade 20A by an additional three consecutive mutations: G‐to‐A at position 28 881, G‐to‐A at 28 882 and G‐to‐C at 28 883. The September sample (EPI_ISL_677634) also shows numerous downstream mutations within lineage 20B, including five non‐synonymous mutations in Orf1a‐Nsp3 (C6027T: P1921L), Orf1a‐Nsp6 (C11514T: T3750I), the Spike glycoprotein (C20703T: V3G and C21575T: L5F) and Orf3a (G25599T: W69C) (Table S3).

## 4 DISCUSSION

Like many countries in sub‐Saharan Africa, Madagascar quickly imposed a border closure and a lockdown of the capital city following the first detected case of COVID‐19. To limit the spread and contain the epidemic, the MPH commissioned testing of all passengers arriving from affected countries (mainly Europe) from 12 March to the date of closure of air‐traffic (20 March 2020). All identified and reachable air‐passengers that arrived in Madagascar during that period were sampled and tested independently of clinical signs. Some of them were quarantined upon arrival, while others were tested retrospectively after returning home for several days with their relatives. Despite attempts to prevent introductions, the first locally acquired cases were detected on 25 March 2020, suggestive of introductions prior to border closure. Nevertheless, community transmission remained limited until the end of April, with only sporadic cases detected, followed by strong measures to isolate patients, and trace and test all contacts. Unfortunately, in May 2020, an increasing number of cases from several clusters were detected in Toamasina, the second highest populated city of the country. The outbreak started initially among the several hundred employees of a large mining company that operates in the city. Despite efforts to contain the outbreak, the virus rapidly spread throughout the city and neighbouring region. This outbreak lasted for 8 weeks (from 27 April to 21 June 2020). Following this major outbreak in Toamasina, cases began to rise in the capital of Antananarivo during the first week of June. Sequence data are not yet resolved sufficiently to determine if the outbreak affecting Antananarivo was a consequence of individuals arriving from Toamasina despite regional containment measures or if it resulted from low‐level circulation within Antananarivo following the first introductions in March. As May to September marks the dry, cold season in the Madagascar highlands, climate may have also played a role in amplifying the epidemic; indeed, previous studies have shown that active circulation of influenza viruses in Madagascar and particularly in Antananarivo is observed between May to September during the dry and cold season in the highlands.^23^, ^24^, ^25^ Further sequencing of SARS‐CoV‐2 isolates will be critical to “tracing” the spread of these two different outbreaks.

Overall, the total number of laboratory‐confirmed cases of COVID‐19 in Madagascar as of 20 September 2020 (16,020, a third of which were detected in part with this study) remained low per inhabitant, when compared to Europe and the Americas.^26^ Within Africa, Madagascar is among the ten countries reporting the highest number of cases of COVID‐19 but is still reporting far fewer cases than the northern African countries, as well as South Africa.^26^, ^27^ Several reasons could explain this result. First, almost 65% of Madagascar's inhabitants live in rural settings,^8^ and the population is, on average, very young (median age = 20.3 years). In our study, SARS‐CoV‐2 infected patients aged less than 20 years represented only 9.4% of all positive cases. This particularity may have limited the spread of COVID‐19 as suggested by the modelling study conducted by Diop *et al*.^28^ Secondly, it is possible that the total number of confirmed cases of COVID‐19 in Madagascar is underestimated and/or underreported due to several factors, including (i) the testing capacity of labs that could not exceed 1,000 tests/day, (ii) insufficient staff to conduct efficient contact tracing and (iii) behavioral resistance to healthcare seeking in the population. Limited healthcare seeking behaviour often presents challenges to efforts to estimate the burden of diseases in sub‐Saharan and other low‐income countries.^29^, ^30^ Resistance to seeking health care can have many drivers, but recent studies have shown a reduction in patient presentation in clinics or hospitals during the COVID‐19 pandemic and associated lockdown.^31^, ^32^, ^33^ An ongoing serological survey among blood donors in Madagascar should be able to address the true burden of COVID‐19.

For future monitoring of SARS‐CoV‐2 circulation, WHO has recently recommended that countries extend the Influenza Surveillance System (ISS) to include COVID‐19.^13^ In Madagascar, an effective ISS has been in place for decades and was used effectively to detect and monitor the last pandemic virus A/H1N1pdm09 in Madagascar.^11^, ^12^ Although the ISS was disrupted during the first few weeks of the COVID‐19 epidemic, due to a lack of personal protective equipment for clinicians and their excessive workload, it was rapidly reinstated and has been used thereafter for effective monitoring of SARS‐CoV‐2 circulation in the Madagascar community. Indeed, 3.7% (207/5,553) of all COVID‐19 cases considered in this study were sampled in the ISS. The ISS was also responsible for identification of the first cases of COVID‐19 in some of Madagascar's cities (ie Antsirabe and Toamasina). Interestingly, both positivity rate and total case number for COVID‐19 in the ISS peaked in July 2020, mirroring the peak witnessed in the national data published by the MPH, which reported a peak of 614 daily cases on July 22.^5^ This finding demonstrates the importance and public value of the WHO recommendation to extend national ISS to include COVID‐19, as emphasized in a recent publication.^13^, ^34^

In our study, we found that the median age of positive COVID‐19 cases in Madagascar was 39 years (IQR: 28‐52 years), with most positive patients aged 20 years and older (90.6%). Nevertheless, the positivity rates increased with age. These findings are similar to those previously observed in other low‐income countries like Algeria, Nigeria and Pakistan,^35^, ^36^, ^37^ but show an average infection distribution that is younger than that previously reported from Wuhan (median age = 59 years).^38^ These differences likely reflect both the younger age structure of the Madagascar population (median age = 20.3 years) and the national strategy aimed at testing both patients presenting to clinics with pneumonia, as well as travellers and contacts regardless of symptoms at the time of sampling. Indeed, 60% of positive cases in our study declared no symptoms at the time of sampling. This proportion of patient may have impact on the spread of the epidemic. Children under 15 years of age represented only 4.4% of all positive SARS‐CoV‐2 cases in Madagascar, consistent with global patterns showing lower infection rates in children, and in contrast to previously described patterns of respiratory virus circulation in Madagascar.^24^, ^25^, ^39^, ^40^

Regarding clinical signs, although symptoms of COVID‐19 are considered to be non‐specific, the five most common clinical manifestations (fever, cough, weakness, headache and runny nose) were significantly associated with SARS‐CoV‐2 infection in our study. Indeed, a recently published article from one Antananarivo hospital leveraged this finding to adopt a clinical screening score used to assess the probability of COVID‐19 infection.^41^

Initial sequence data indicate multiple introduction events of SARS‐CoV‐2 to Madagascar, with sequences derived from a largely Asian clade of the virus sourcing the initial outbreak in Toamasina, and sequences derived from at least two primarily European clades of the virus sourcing the subsequent outbreak in the capital city of Antananarivo. Notably, the initial SARS‐CoV‐2 sequences from Toamasina lacked the D614G mutation that has been shown to enhance SARS‐CoV‐2 transmissibility,^42^ while those sequences from Antananarivo contained it. Further sequencing of additional isolates from these disparate introduction events in Madagascar should allow us to compare the persistence, duration and transmission capacity of these different SARS‐CoV‐2 lineages. It is important to highlight that the G204R mutation found in both of the later epidemic sequences (EPI_ISL_625456 and EPI_ISL_677634) may affect the binding of primers used in the China CDC assay for N‐gene detection.^16^ This information will need to be addressed in ongoing surveillance. It highlights the need to utilise multiple genetic targets for PCR testing, as well as the importance of periodic genome sequencing of circulating strains to quickly identify any mutation that might affect molecular testing.

Our study has some limitations. Beginning in May 2020, the Madagascar MPH decreed that samples from hospitalized patients should also be tested in public laboratories. Subsequent to this decree, other laboratories began to receive samples not included in these analyses. Additionally, during the first month of our current study, we tested mostly international travellers returning from affected areas, as well as their contacts regardless of symptoms. As such, our data do not represent the full spectrum of clinical cases in Madagascar. In contrast, however, these findings underline the importance of asymptomatic transmission for SARS‐CoV‐2. The role of asymptomatic carriers in the epidemiology of the current pandemic is disputed, but recent paper estimates that half of the overall transmission of SARS‐CoV‐2 are coming from infected patient that present no symptoms.^43^, ^44^ Ongoing studies are currently collecting information on a follow‐up cohort of infected patients and their households and contacts to elucidate more thoroughly the epidemiology of this first wave of SARS‐CoV‐2 in Madagascar. If the role of asymptomatic in the transmission is confirmed, health authorities should consider these population of infected individuals to contain the spread of the virus by increasing the proportion of asymptomatic patients being tracked and screened.

In conclusion, despite strong interventions to prevent and contain the spread of the COVID‐19 epidemic in Madagascar (including lockdowns, curfews, travel restrictions and social distancing), Madagascar was unable to avoid the introduction and the spread of the virus in the country. Nonetheless, these strategies may have helped delay the onset of the epidemic and allowed the MPH to prepare for the response, especially in health districts with limited infrastructure for severe case management. Indeed, the strategy to test specimen from passengers, contacts of confirmed case regardless of symptoms allowed the detection of a substantial proportion of SARS‐CoV‐2 cases (respectively 11.2% and 17.5%) and conduct the health authorities to the isolate those individuals that could have spread the virus in the community. It is yet too early to estimate the true impact of prevention measures taken at both the national and local level on the spread of COVID‐19 in Madagascar. Further work is needed to determine if various interventions effectively delayed the spread of SARS‐CoV‐2 in country or successfully reduced the magnitude of the epidemic. Ongoing studies (seroprevalence surveys, first few hundred cases and contact analyses, and genomic epidemiology) will support efforts to estimate the burden of disease, various epidemiological parameters (eg R0, clinical attack rate, immune response…), and underreporting of cases and inform public health strategies critical to avoiding or reducing the impact of subsequent waves of infection on the health systems and the economy of a country with limited resources.

## CONFLICT OF INTEREST

All authors declare that they have no commercial or other associations that may pose a conflict of interest.

## AUTHOR CONTRIBUTION

**Rindra Vatosoa Randremanana:** Conceptualization (lead); Data curation (supporting); Formal analysis (equal); Funding acquisition (lead); Investigation (equal); Methodology (lead); Supervision (lead); Writing‐original draft (equal); Writing‐review & editing (lead). **Soa‐Fy Andriamandimby:** Data curation (supporting); Formal analysis (supporting); Methodology (supporting); Supervision (equal); Validation (equal); Writing‐review & editing (equal). **Jean Marius Rakotondramanga:** Data curation (lead); Formal analysis (equal); Methodology (supporting); Software (equal); Validation (equal); Visualization (equal); Writing‐original draft (supporting); Writing‐review & editing (supporting). **Norosoa Harline Razanajatovo:** Data curation (supporting); Formal analysis (equal); Methodology (equal); Supervision (supporting); Validation (equal); Writing‐review & editing (supporting). **Reziky Tiandraza Mangahasimbola:** Software (lead); Supervision (supporting); Writing‐review & editing (supporting). **Tsiry Hasina Randriambolamanantsoa:** Formal analysis (equal); Investigation (supporting); Methodology (supporting); Validation (supporting); Writing‐review & editing (supporting). **Hafaliana Christian Ranaivoson:** Data curation (equal); Formal analysis (equal); Writing‐review & editing (supporting). **Harinirina Aina Rabemananjara:** Formal analysis (equal); Investigation (supporting); Methodology (supporting); Writing‐review & editing (supporting). **Iony Razanajatovo:** Formal analysis (supporting); Methodology (supporting); Supervision (equal); Validation (equal). **Richter Razafindratsimandresy:** Supervision (equal); Validation (equal); Writing‐review & editing (supporting). **Joelinotahina Hasina Rabarison:** Data curation (equal); Formal analysis (supporting); Investigation (equal); Supervision (supporting); Writing‐review & editing (supporting). **Cara E Brook:** Data curation (supporting); Formal analysis (supporting); Funding acquisition (supporting); Software (supporting); Visualization (supporting); Writing‐review & editing (equal). **Fanjasoa Rakotomanana:** Software (equal); Visualization (equal); Writing‐review & editing (supporting). **Roger Mario Rabetombosoa:** Investigation (supporting); Resources (supporting); Supervision (supporting). **Helisoa Razafimanjato:** Data curation (supporting); Formal analysis (equal); Methodology (supporting); Validation (equal). **Vida Ahyong:** Data curation (supporting); Formal analysis (supporting); Software (supporting); Validation (supporting). **Vololoniaina Raharinosy:** Formal analysis (supporting); Methodology (supporting); Validation (supporting). **Vaomalala Raharimanga:** Data curation (supporting); Investigation (supporting); Supervision (supporting). **Sandratana Jonhson Raharinantoanina:** Formal analysis (supporting); Investigation (lead); Supervision (supporting); Validation (supporting). **Mirella Malala Randrianarisoa:** Data curation (supporting); Investigation (supporting). **Barivola Bernardson:** Investigation (supporting); Supervision (supporting). **Laurence Randrianasolo:** Investigation (equal); Project administration (equal); Resources (equal); Supervision (supporting). **Léa Bricette Nirina Randriamampionona:** Investigation (equal); Project administration (supporting); Resources (equal); Supervision (supporting); Validation (supporting). **Cristina M. Tato:** Data curation (supporting); Funding acquisition (supporting); Project administration (supporting); Software (supporting); Validation (supporting); Writing‐review & editing (supporting). **Joseph L. DeRisi:** Data curation (supporting); Funding acquisition (supporting); Resources (supporting); Validation (supporting); Writing‐review & editing (equal). **Manuela Christophère Vololoniaina:** Resources (lead); Supervision (equal); Validation (supporting); Writing‐review & editing (supporting). **Fidiniaina Mamy Randriatsarafara:** Investigation (equal); Resources (lead); Supervision (equal); Writing‐review & editing (supporting). **Zely Arivelo Randriamanantany:** Conceptualization (supporting); Investigation (lead); Project administration (equal); Resources (lead); Supervision (supporting); Writing‐review & editing (supporting). **Jean‐Michel Heraud:** Conceptualization (lead); Data curation (equal); Formal analysis (equal); Funding acquisition (equal); Investigation (equal); Methodology (equal); Project administration (lead); Resources (lead); Supervision (equal); Validation (supporting); Writing‐original draft (lead); Writing‐review & editing (lead).

### PEER REVIEW

The peer review history for this article is available at https://publons.com/publon/10.1111/irv.12845.

## Supporting information

Supplementary Material
