# Supplementary material for: The COVID‐19 epidemic in Madagascar: clinical description and laboratory results of the first wave, march‐september 2020
Source: Influenza Other Respir Viruses. 2021 Feb 15;15(4):457–68. doi: 10.1111/irv.12845 (PMC8013501; doi:10.1111/irv.12845)
Supplement: Supplementary file 1 — Supplementary Material [file IRV-15-457-s001.docx]

**Supplementary Methods: Generating full genome sequences from strains detected in Madagascar:**

We sequenced the genomes of six SARS-CoV-2 positive samples to elucidate the phylogenetic history of pathogen spread in Madagascar. NGS libraries for the first two sequences (EPI_ISL_508862 and EPI_ISL_508863) were prepared from viral RNA extracted from the original NP swab sample, following a Metagenomic Sequencing with Spike Primer Enrichment (MSSPE) protocol, and using the NEBNext® Ultra II RNA Library Prep Kit (New England Biolabs, Ipswich, MA, USA) (1). Because of differences in the Ct value of the original sample (Ct=15.86 in the case of EPI_ISL_508862 and Ct=20.89 in the case of EPI_ISL_508863), we used different concentrations of random hexamer (RH) and SARS-CoV-2 primer (SC2) for the two different samples, respectively, RH:SC2=1µM:10µM and RH:SC2=10µM:100µM. Library size and concentration were determined using a 4150 TapeStation system (Agilent, Santa Clara, CA, USA), and the resulting libraries were sequenced on an Illumina iSeq100 instrument (Illumina, San Diego, CA, USA) using 150 nucleotide paired-end sequencing. Resulting raw fastq files were uploaded to the SARS-CoV-2 consensus pipeline on the IDseq portal, a cloud-based, open-source bioinformatics platform (<https://idseq.net>), to generate composite fasta files. Ambiguous basepairs and blanks (N) in the consensus genome were subsequently resolved via manual editing in Geneious Prime 2020.0.5 after mapping the raw fastq files to the Wuhan reference genome (hCov-19/Wuhan/WIV04/2019) and examining any contested nucleotides.

Genomes of the remaining four samples (EPI_ISL_677635, EPI_ISL_677636, EPI_ISL_677634, and EPI_ISL_625456) were obtained in a similar manner, though library preparation were this time based on Josh. Quick V3 Artic protocol for Amplicon sequencing of SARS-CoV-2 with few deviations (2). After obtaining SARS-CoV-2 amplicons, libraries were prepped with NEBNext Ultra II FS DNA Library Prep Kit for Illumina (New England Biolabs, Ipswich, MA, USA). As before, library size and concentration were determined using a 4150 TapeStation system, and resulting libraries were sequenced on an Illumina iSeq100 instrument using 150 nucleotide paired-end sequencing. Raw fastq files were again uploaded to the SARS-CoV-2 consensus pipeline on the IDseq portal, and ambiguous basepairs and blanks (N) in the consensus genome were resolved via manual editing in Geneious Prime 2020.0.5.

After consensus genomes for all six samples were verified, sequences were uploaded to the public repository, GISAID.org, from which developers of the open source phylogenetics platform, Nextstrain.org, automatically pull all available SARS-CoV-2 sequences for global comparison and visualization (3). We downloaded the Nextstrain.org SARS-CoV-2 global timetree on 14 December 2020 for visualization of our six Madagascar genomes in Supplementary Figure. Nextstrain.org uses a Python-based framework for phylodynamic analysis and a Maximum Likelihood approach for efficient timetree computation (4).

**Supplementary Table 1: Number of SARS-CoV-2 laboratory-confirmed cases and positivity rates per group of patients (passengers, suspected case, and contact of confirmed cases), Madagascar 18 March to 18 September 2020.** Passengers were identified as individuals with an history of travel as defined in the materials and methods (cf. Study subject and specimen collection). As the variable screening/convenient specimens was not indicated in the CRF, all individuals sampled at screening-test clinics of for conveniency were entered has suspected case.

| **Group of patients** | **N** | **SARS-CoV-2** | **%** |
| --- | --- | --- | --- |
| **Passengers** | 878 | 98 | 11.2 |
| **Suspected** | 16,219 | 3,819 | 23.5 |
| **Contact** | 9,318 | 1,636 | 17.6 |
| **Total** | 26,415 | 5,553 | 21.0 |

**Supplementary Table 2: Distribution of clinical signs among laboratory confirmed SARS-CoV-2 patients presenting symptoms at time of sampling (N=5,472).**

| **Clinical signs** | **N** | **%** |
| --- | --- | --- |
| **Cough** | 1,488 | 27.2 |
| **Fever** | 1,025 | 18.7 |
| **Weakness** | 804 | 14.7 |
| **Runny Nose** | 729 | 13.3 |
| **Headache** | 717 | 13.1 |
| **Arthralgia** | 516 | 9.4 |
| **Myalgia** | 501 | 9.2 |
| **Sore throat** | 354 | 6.5 |
| **Dyspnoea** | 311 | 5.7 |
| **Pain** | 216 | 3.9 |
| **Diarrhoea** | 168 | 3.1 |
| **Vomiting** | 139 | 2.5 |
| **Abdominal pain** | 77 | 1.4 |
| **Wheezing** | 38 | 0.7 |
| **Haemoptysis** | 35 | 0.6 |
| **Conjunctivitis** | 26 | 0.5 |
| **Otalgia** | 17 | 0.3 |
| **Intercostal Indrawing** | 13 | 0.2 |
| **Mental Confusion** | 13 | 0.2 |
| **Convulsion** | 6 | 0.1 |
| **Rash** | 6 | 0.1 |
| **Ulceration** | 6 | 0.1 |
| **Adenopathy** | 3 | 0.1 |
| **Haemorrhage** | 2 | 0.0 |

**Supplementary Figure 1: Number of SARS-CoV-2 laboratory-confirmed cases and positivity rates per age group, Madagascar 18 March to 18 September 2020.**

**Supplementary Figure 2: Phylogenetic three showing the global positioning of strains detected in Madagascar from March to September 2020.** Raw Newick file of the latest global analysis of SARS-CoV-2 genomes was downloaded from Nextstrain.org on 14 December 2020, then visualized in R using the package, ‘ggtree’ (5). The Nextstrain.org subsample of 3527 genomes are shown as phylogeny tips coloured by region of sample collection. The nodes of origin for the major phylogenetic clades in circulation (19A, 19B, 20A, 20B, 20C) are labelled, and all six Madagascar sequences are highlighted as large, named red circles.


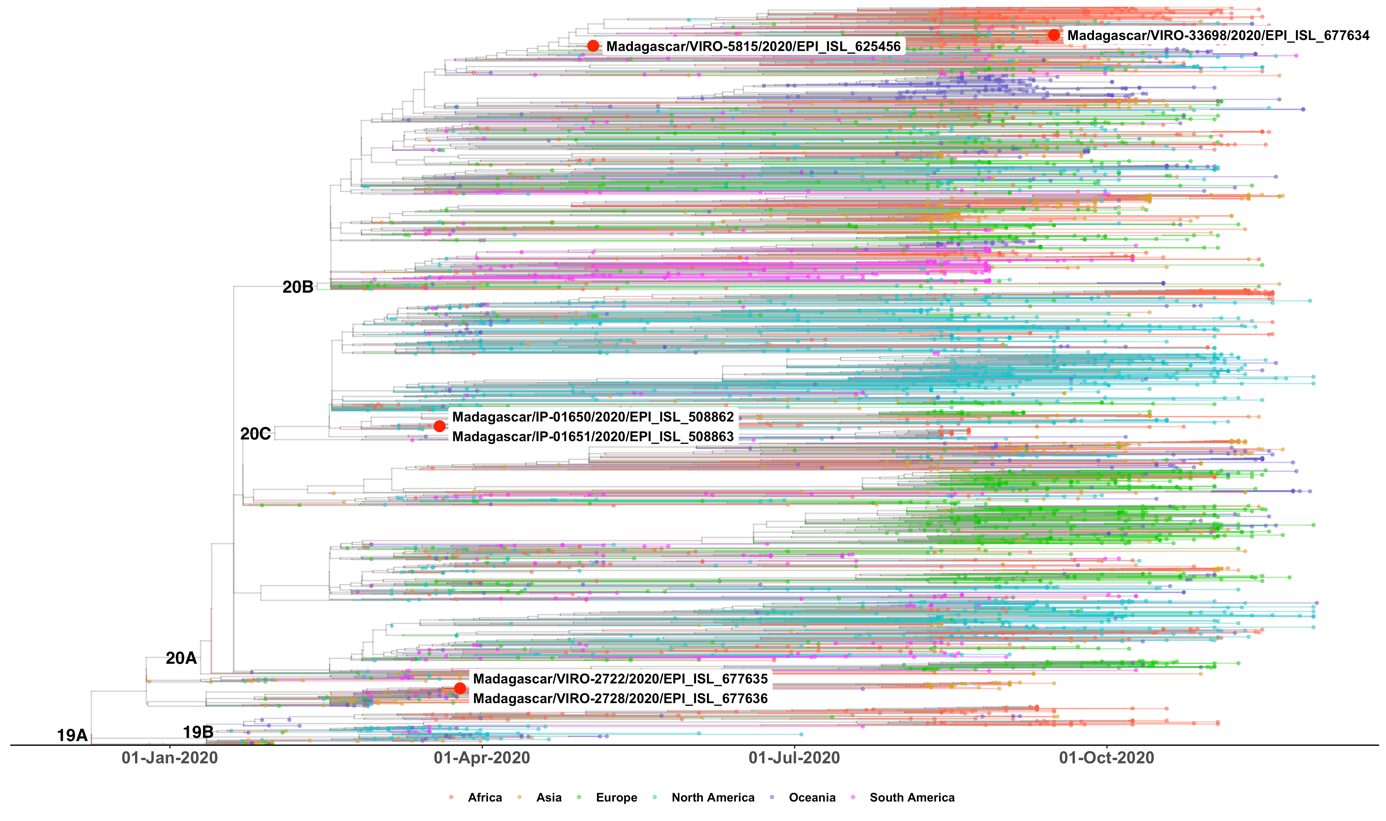


**Supplementary Table 3: Genetic characteristic (clades, substitutions, and amino acid changes) of full genome sequences of SARS-CoV-2 viruses detected in Madagascar from March to September 2020.**

| **GISAID EPI Number** | **Date of Collection** | **Location (City)** | **Introduced /**  **Local^†^** | **Clade (Nextstrain)** | **Nucleotide Substitutions** | **Amino Acid Changes^‡^** | **PCR Primer Changes^§^** |
| --- | --- | --- | --- | --- | --- | --- | --- |
| EPI_ISL_508862 | 20/03/20 | Antananarivo | Introduced (France) | 20A | C241T, C2416T, C3037T, C5884T, C14408T, A23403G, G25563T | ORF1b: P314L  S: **D614G**  ORF3a: Q57H | - |
| EPI_ISL_508863 | 22/03/20 | Fenoarivo | Introduced (France) | 20A | C241T, C2416T, C3037T, C5884T, C14408T, A23403G, G25563T | ORF1b: P314L  S: **D614G**  ORF3a: Q57H | - |
| EPI_ISL_677635 | 26/03/20 | Toamasina | Introduced (Philippines) | 19A | G1268A, C6312A, G11083T, C13730T, C19524T, C23929T, C28311T | ORF1a: D335N; T2016K; L3606F  ORF1b: A88V  N: P13L  ORF9b: P10S | USCDC (N1)  P: C28311T |
| EPI_ISL_677636 | 26/03/20 | Toamasina | Introduced (Philippines) | 19A | G1268A, C6312A, G11083T, C13730T, C19524T, C23929T, C28311T | ORF1a: D335N; T2016K; L3606F  ORF1b: A88V  N: P13L  ORF9b: P10S | USCDC (N1)  P: C28311T |
| EPI_ISL_625456 | 04/05/20 | Toamasina | Local | 20B | C241T, C3037T, C14408T, A23403G, G28881A, G28882A, G28883C | ORF1b: P314L  S: **D614G**  N: R203K; G204R | ChinaCDC (N)  F: G28881A; G28882A; G28883C |
| EPI_ISL_677634 | 16/09/20 | Antananarivo | Local | 20B | C241T, C1609T, C3037T, C6027T, C10702T, C11514T, A11782G, C14408T, C20703T, T21570G, C21575T, A23403G, T25473C, G25599T, G28881A, G28882A, G28883C | ORF1a: P1921L; T3750I  ORF1b: P314L  S: V3G; L5F; **D614G**  ORF3a: W69C  N: R203K; G204R | ChinaCDC (N)  F: G28881A; G28882A; G28883C |

^†^: The countries mentioned for introduced strains are based on location where patients were originated from before arriving in Madagascar. This does not exclude the possibility that infection was acquired during his travel to Madagascar. Local= local transmission

^‡^: ORF= Open Reading Frame; N=Nucleocapsid; S= Spike glycoprotein.

^§^: Type of primer changes: “Protocol Name, (Target), Position of the substitution in the primer”. (P=Probes, F=Forward primer)

**Supplementary references:**

1. Deng X, Achari A, Federman S, Yu G, Somasekar S, Bartolo I, et al. Metagenomic sequencing with spiked primer enrichment for viral diagnostics and genomic surveillance. Nat Microbiol. 2020;5(3):443-54.

2. Quick J. nCoV-2019 sequencing protocol v3 (LoCost). protocols.io 2020 [Available from: <https://protocols.io/view/ncov-2019-sequencing-protocol-v3-locost-bh42j8ye>.

3. Hadfield J, Megill C, Bell SM, Huddleston J, Potter B, Callender C, et al. Nextstrain: real-time tracking of pathogen evolution. Bioinformatics. 2018;34(23):4121-3.

4. Sagulenko P, Puller V, Neher RA. TreeTime: Maximum-likelihood phylodynamic analysis. Virus Evol. 2018;4(1):vex042.

5. Yu G, Smith DK, Zhu H, Guan Y, Lam TTY, McInerny G. GGTREE : an R package for visualization and annotation of phylogenetic trees with their covariates and other associated data. Methods in Ecology and Evolution. 2016;8(1):28-36.
